# Evaluation of the efficacy and safety of cannabidiol-rich cannabis extract in children with autism spectrum disorder: randomized, double-blind, and placebo-controlled clinical trial

**DOI:** 10.47626/2237-6089-2021-0396

**Published:** 2024-02-28

**Authors:** Estácio Amaro da Silva Junior, Wandersonia Moreira Brito Medeiros, João Paulo Mendes dos Santos, João Marçal Medeiros de Sousa, Filipe Barbosa da Costa, Katiúscia Moreira Pontes, Thaís Cavalcanti Borges, Carlos Espínola Neto Segundo, Ana Hermínia Andrade e Silva, Eliane Lima Guerra Nunes, Nelson Torro Alves, Marine Diniz da Rosa, Katy Lísias Gondim Dias de Albuquerque

**Affiliations:** 1 Programa de Pós-Graduação em Neurociência Cognitiva e Comportamento Departamento de Psicologia UFPB João Pessoa PB Brazil Programa de Pós-Graduação em Neurociência Cognitiva e Comportamento, Departamento de Psicologia, Universidade Federal da Paraíba (UFPB), João Pessoa, PB, Brazil.; 2 UFPB João Pessoa PB Brazil UFPB, João Pessoa, PB, Brazil.; 3 Departamento de Estatística UFPB João Pessoa PB Brazil Departamento de Estatística, UFPB, João Pessoa, PB, Brazil.; 4 SBEC São Paulo SP Brazil Sociedade Brasileira de Estudos da Cannabis Sativa (SBEC), São Paulo, SP, Brazil.; 5 Departamento de Psicologia UFPB João Pessoa PB Brazil Departamento de Psicologia, UFPB, João Pessoa, PB, Brazil.; 6 Departamento de Fonoaudiologia UFPB João Pessoa PB Brazil Departamento de Fonoaudiologia, UFPB, João Pessoa, PB, Brazil.; 7 Departamento de Fisiologia e Patologia UFPB João Pessoa PB Brazil Departamento de Fisiologia e Patologia, UFPB, João Pessoa, PB, Brazil.

**Keywords:** Autism spectrum disorder, child behavior, clinical trial, cannabis, cannabidiol

## Abstract

**Objective:**

Autism spectrum disorder (ASD) is characterized by persistent deficits in social communication and social interaction and by restricted and repetitive patterns of behavior. Some studies have shown that substances derived from *Cannabis sativa* improve the quality of life of children with ASD without causing serious adverse effects, thus providing an alternative therapeutic option. The objective of this study was to evaluate the efficacy and safety of a cannabis extract rich in cannabidiol (CBD) in children with ASD.

**Methods:**

In this randomized, double-blind, placebo-controlled clinical trial, 60 children, aged from 5 to 11 years, were selected and divided into two groups: the treatment group, which received the CBD-rich cannabis extract, and the control group, which received the placebo. They both used their respective products for a period of 12 weeks. Statistical analysis was done by two-factor mixed analysis of variance (two-way ANOVA).

**Results:**

Significant results were found for social interaction (F_1,116_ = 14.13, p = 0.0002), anxiety (F_1,116_ = 5.99, p = 0.016), psychomotor agitation (F_1,116_ = 9.22, p = 0.003), number of meals a day (F_1,116_ = 4.11, p = 0.04), and concentration (F_1,48_ = 6.75, p = 0.01), the last of which was only significant in mild ASD cases. Regarding safety, it was found that only three children in the treatment group (9.7%) had adverse effects, namely dizziness, insomnia, colic, and weight gain.

**Conclusion:**

CBD-rich cannabis extract was found to improve one of the diagnostic criteria for ASD (social interaction), as well as features that often co-exist with ASD, and to have few serious adverse effects.

## Introduction

According to the Diagnostic and Statistical Manual of Mental Disorders (DSM-5), autism spectrum disorder (ASD) is a neurodevelopmental disorder characterized by persistent deficits in social communication and social interaction, in multiple contexts, and by the presence of restricted and repetitive patterns of behavior, interests, or activities. The DSM-5 also adopts ASD severity level as a specifying criterion, which varies according to the need for support: mild (needs support), moderate (needs substantial support), or severe (needs very substantial support).^1^

There are no treatments proven to target the core features of ASD. Existing treatments are solely symptomatic, mainly aiming to reduce aggressiveness and psychomotor agitation symptoms and usually using psychotropic medications to effect these behavioral changes. In order to help patients affected by this disorder, there is great interest in discovering new therapeutic options to overcome the ineffectiveness of some of the conventional psychotropic drugs used to treat ASD, or even to enable their suspension, reducing the adverse effects associated with these drugs.^2^

Endocannabinoids are substances that are part of the endocannabinoid system (ECS) that are key modulators of socioemotional responses, cognition, seizure susceptibility, nociception, and neuronal plasticity and all of these responses are altered in autism.^3,4^ Phytocannabinoids, mainly cannabidiol (CBD) and tetrahydrocannabinol (THC), present in several subspecies of the Cannabis genus, have been widely studied as a potential therapeutic alternative for treatment of symptoms associated with ASD, because they activate cannabinoid receptors present in the central nervous system, alleviating some symptoms associated with autism.^5^

Even though cannabis is becoming a topic of great interest among scientists, it is still difficult to find clinical trials with humans due to the restrictions and legal issues involving the plant.^6^ Studies using cannabis for treatment of difficult-to-control epilepsy have found that, in addition to seizure reduction, there were also improvements in behavior and social interaction in children who had ASD as a comorbidity.^7^

In light of the above, the purpose of this research is to evaluate the efficacy of CBD-rich cannabis extract in children with ASD, monitoring aspects from the DSM-5 diagnostic criteria (social interaction, speech, and stereotypes) and other aspects that often coexist with ASD (aggressiveness, psychomotor agitation, impaired concentration, eating disorders, sleep disorders, and anxiety), as well as to assess the tolerability and safety of the therapeutic adjuvant.

## Method

This study was a randomized, double-blind, placebo-controlled, 12-week clinical trial following the CONSORT recommendations. Initially, G Power software was used to calculate the sample size based on studies by Handen et al.^8^ with children with ASD who took donepezil. An alpha of 0.05 and power of 0.80 were used. The calculation showed that 62 subjects would be needed (31 per group).

This study included children aged from 5 to 11 years who lived in the state of Paraíba, Brazil, or in neighboring states (Pernambuco and Rio Grande do Norte), who had a medical diagnosis of ASD, regardless of whether they had mild, moderate, or severe levels of ASD impairment, and whose caregivers signed the informed consent form. This age group was selected because children aged from 5 to 11 years exhibit greater similarity in brain development, making the group more homogeneous for analysis of the results. In addition, children older than 5 years would be more likely to have developed verbal language and be better able to respond during neuropsychological testing. For these reasons, we chose this age group to participate in this study. Children who had comorbidities such as diabetes mellitus, hypertension, autoimmune diseases, or refractory epilepsy or who had used a cannabis product in the last two months before starting the study were excluded.

To recruit the sample, the study was publicized widely through autism support institutions, with informative lectures and posts on WhatsApp and social media (Facebook and Instagram). Thereafter, those interested in the study registered on a website created exclusively for this clinical trial and filled out the sociodemographic questionnaire and the Childhood Autism Rating Scale (CARS) screening instrument.^9^ The cut-off score is 15 points and CARS results were also used to assess severity. After applying the eligibility criteria, the researchers contacted caregivers to provide further explanations about the research.

After recruitment, the 64 selected children were randomized and stratified by severity. The randomization was conducted using the True Random Number Service, available at www.random.org. Any researchers who had direct contact with the patients were blinded to the treatment provided, except for one pharmacy student, who was responsible for delivering the vials to the researching physician weekly. Finally, baseline evaluations were scheduled with all children participating in this study and performed by the same child and adolescent psychiatrist responsible for the trial, to whom the products for the trial were also delivered. Caregivers were instructed to always start with a dose of three drops every 12 hours, preferably during a fasting period and with an interval of at least 1 hour before or after use of psychotropic medication, especially antipsychotics, according to the protocol suggested by the product supplier, since it was a plant extract. The starting dose of CBD-rich cannabis extract used in this study was six drops daily, increased by two drops daily twice a week, if necessary, up to a maximum dose of 70 drops daily.

This clinical study was conducted in the outpatient sector, on the campus of the Hospital Universitário Lauro Wanderley, Universidade Federal da Paraíba (UFPB), which is located in João Pessoa, Paraíba. This research was approved by the institutional ethics review board at the UFPB Centro de Ciências da Saúde, under CAAE number 89392518.4.0000.5188. It is registered on the Brazilian Registry of Clinical Trials (ReBEC) under number 10743.

The product used was CBD-rich cannabis extract at a concentration of 0.5% (5 mg/mL), in the ratio of 9CBD:1THC, supplied by the Associação Brasileira de Apoio Cannabis Esperança (ABRACE). The extract used throughout the clinical trial was from the same batch, in order to ensure the same phytochemical and pharmacobotanical characteristics during production of the extract. The CBD-rich cannabis extract and the placebo product without it had the same consistency, color, odor, and other organoleptic characteristics, making it impossible for patients or the multidisciplinary team accompanying them to differentiate between the two.

To evaluate the effectiveness of the treatment, we used a semi-structured interview prepared by the authors containing questions related to ASD symptoms and the Autism Treatment Evaluation Checklist (ATEC),^10^ which were administered to and answered by caregivers before and after the clinical trial. The number of daily meals was reported by the child’s caregiver during the psychiatric consultation after they had answered the semi-structured interview questionnaire. In order to assess safety, before starting the study, all children underwent a laboratory evaluation, including kidney and liver function tests, as well as complete blood count and fasting glucose levels.

All analyses were performed using R version 4.0.2, which is free software available at https://www.r-project.org/. A 5% significance level was adopted for all analyses. Statistical analysis was performed using the mixed variance test for two factors (two-way ANOVA). In cases in which the null hypothesis was rejected, Tukey’s multiple comparisons post-hoc test was applied to identify which groups had significant differences. As there is no non-parametric technique available in the literature regarding mixed analysis of variance for two factors, a simple non-parametric analysis was used for each factor individually, in order to support the results obtained by the parametric technique. The treatment and control groups and before and after data were compared using the Wilcoxon test for independent and dependent samples respectively.

## Results

### Sociodemographic analysis of participants’ parents

A range of different sociodemographic variables regarding the parents were evaluated, including: age; education; whether they had another child, and whether they had other children on the autism spectrum; whether father and/or mother went to work away from the home; whether one of the parents had had to stop working because the child was diagnosed with ASD; and parents’ marital status (Table 1).

**Table 1:** Sociodemographic data on the parents of children with autism spectrum disorder (ASD)

Variable (parents)	Treatment group (n = 31)	Control group (n = 29)	Total (n = 60)
Mother’s age (at conception)	29.00 (29.00) ± 6.09	30.00 (30.00) ± 6.85	29.46 (30.00) ± 6.42
Father’s age (at conception)	34.05 (34.00) ± 6.69	32.10 (31.00) ± 5.67	33.10 (33.00) ± 6.21
Mother’s education			
Incomplete elementary	1 (3.57)	2 (7.69)	3 (5.56)
Complete elementary	0 (0.00)	1 (3.85)	1 (1.85)
Incomplete secondary	2 (7.14)	0 (0.00)	2 (3.70)
Complete secondary	9 (32.14)	7 (26.92)	16 (29.63)
Incomplete higher	4 (14.29)	6 (23.08)	10 (18.52)
Complete higher	6 (21.43)	4 (15.38)	10 (18.52)
Postgraduate	6 (21.43)	6 (23.08)	12 (22.22)
Father’s education			
Incomplete elementary	0 (0.00)	1 (5.56)	1 (2.63)
Complete elementary	1 (5.00)	1 (5.56)	2 (5.26)
Incomplete secondary	3 (15.00)	6 (33.33)	9 (23.68)
Complete secondary	2 (10.00)	2 (11.11)	4 (10.53)
Incomplete higher	8 (40.00)	4 (22.22)	12 (31.58)
Complete higher	6 (30.00)	4 (22.22)	10 (26.32)
Other children			
No	11 (35.48)	10 (34.48)	21 (35.00)
Yes	14 (45.16)	14 (48.28)	28 (46.67)
Yes and with ASD	6 (19.35)	5 (17.24)	11 (18.33)
Working parents			
No	1 (3.23)	2 (6.90)	3 (5.00)
Yes	20 (64.52)	14 (48.28)	34 (56.67)
One of the parents had to stop	10 (32.26)	13 (44.83)	23 (38.33)
Father or mother’s marital status			
Single	8 (25.81)	6 (20.69)	14 (23.33)
Married	15 (48.39)	17 (58.62)	32 (53.33)
Divorced	8 (25.81)	5 (17.24)	13 (21.67)
Other	0 (0.00)	1 (3.45)	1 (1.67)

Qualitative variables: n (%); quantitative variables: average (median) ± standard deviation.

### Sociodemographic analysis of the children

In general, there were no significant differences between the treatment and control groups for the sociodemographic variables evaluated in the children with ASD who participated in this study. However, it is important to observe whether the child was receiving any professional healthcare intervention for ASD (public, private, or mixed) and the professional(s) responsible for this care (occupational therapist, physical therapist, speech therapist, psychologist, psychopedagogue, or others); whether the child was using psychotropic drugs before, during, or after the clinical trial; whether or not the child had food selectivity and whether this eating pattern was modified; the severity of ASD (mild [needs support], moderate [needs substantial support], or severe [needs very substantial support], according to the DSM-5 classification); whether there were adverse effects associated with the use of the product (CBD-rich extract or placebo); the number of drops of the product being taken at the end of the trial, since the increase was gradual and as directed by the researcher; whether the caregiver was in doubt, did not notice, or could not see improvement with the test product at the final consultation, before the researcher or the participants were aware whether the child had been allocated to the treatment or the control group. Finally, because the coronavirus (coronavirus disease 2019 [COVID-19]) pandemic broke out during the clinical trial, the children were isolated at home or could not receive professional attention, causing changes to their routine, which in itself is a disorganizing factor for those with ASD, so the researchers included the participants’ parents’ reports of the effects of isolation on their children’s symptoms in the final evaluation (Table 2).

**Table 2:** Sociodemographic data and information about the children participating in the research

Variable (children)	Treatment group (n = 31)	Control group (n = 29)	Total (n = 60)
Gender			
Male	25 (80.65)	27 (93.10)	52 (86.67)
Female	6 (19.35)	2 (6.90)	8 (13.33)
Age	7.64 (7.00) ± 1.76	7.72 (7.00) ± 1.75	7.68 (7.00) ± 1.74
Child’s education			
Does not attend	2 (6.45)	2 (6.90)	4 (6.67)
Beneath the expected grading	4 (12.90)	6 (20.69)	10 (16.67)
Within the expected grading	25 (80.65)	21 (72.41)	46 (76.67)
Treatment type			
Does not receive treatment	6 (19.35)	7 (24.14)	13 (21.67)
Public	14 (45.16)	9 (31.03)	23 (38.33)
Private	7 (22.58)	11 (37.93)	18 (30.00)
Mixed	4 (12.90)	2 (6.90)	6 (10.00)
Occupational therapy	16 (51.61)	17 (58.62)	33 (55.00)
Physiotherapy	1 (3.23)	1 (3.45)	2 (3.33)
Speech therapy	19 (61.29)	20 (68.97)	39 (65.00)
Psychology	15 (48.39)	17 (58.62)	32 (53.33)
Psychopedagogy	11 (35.48)	12 (41.38)	23 (38.33)
Other treatments	6 (19.35)	2 (6.90)	8 (13.33)
Psychotropics			
Does not use	17 (54.84)	10 (34.48)	27 (45.00)
Used and continued	12 (38.71)	16 (55.17)	28 (46.67)
Used and stopped	1 (3.23)	2 (6.90)	3 (5.00)
Did not use and started	1 (3.23)	1 (3.45)	2 (3.33)
Selective eating			
No	16 (51.61)	17 (58.62)	33 (55.00)
Yes and continued	8 (25.81)	7 (24.14)	15 (25.00)
Yes and stopped	7 (22.58)	5 (17.24)	12 (20.00)
Severity			
Mild	13 (41.94)	13 (44.83)	26 (43.33)
Moderate	16 (51.61)	13 (44.83)	29 (48.33)
Severe	2 (6.45)	3 (10.34)	5 (8.33)
Adverse side effects	4 (12.90)	5 (17.24)	9 (15.00)
Subjective improvement			
No	7 (22.58)	12 (41.38)	19 (31.67)
Unsure	3 (9.68)	7 (24.14)	10 (16.67)
Yes	21 (67.74)	10 (34.48)	31 (51.67)
Number of drops being taken	47.42 (52.00) ± 15.22	40.96 (44.00) ± 18.86	44.30(50.00) ± 17.23
Isolation interfered?			
No	19 (61.29)	19 (65.52)	38 (63.33)
Only initially	4 (12.90)	1 (3.45)	5 (8.33)
Yes	8 (25.81)	9 (31.03)	17 (28.33)

Qualitative variables: n (%); quantitative variables: average (median) ± standard deviation.

### Analysis of the semi-structured interview, ATEC, and CARS

Important variables associated with ASD were assessed using a semi-structured questionnaire. The symptoms evaluated were aggressiveness, psychomotor agitation, concentration, meals (number of meals/day), sleep (number of hours of sleep/day), social interaction with peers, verbal language (speech), anxiety; and repetitive and stereotyped movements (stereotypies). Mean scores were also calculated for the ATEC scale (and its subdivisions: ATEC L, related to language; ATEC S, related to socialization; ATEC P, related to sensory and cognitive perception; ATEC SC, related to health, physical aspects, and behavior; and ATEC T, which is the total sum of the scale) and the CARS scale (Table 3).

**Table 3:** Assessment of different variables in children with autism spectrum disorder (ASD) in the control group and the treatment group

Variable	Treatment group (n = 31)	Control group (n = 29)	p-value
Aggressiveness	0.81 (0.00) ± 1.05	1.39 (1.00) ± 1.36	0.2149
Psychomotor agitation	1.64 (2.00) ± 1.28	2.65 (3.00) ± 1.14	0.00295*
Concentration	1.71 (2.00) ± 1.07	2.96 (3.00) ± 0.86	0.269
Meals	1.32 (0.00) ± 1.90	0.38 (0.00) ± 0.82	0.045^†^
Sleep	0.77 (0.00) ± 1.61	0.28 (0.00) ± 0.59	0.0711
Social interaction	1.68 (2.00) ± 1.01	2.83 (3.00) ± 1.10	0.000268^‡^
Speech	1.32 (1.00) ± 1.42	1.72 (1.00) ± 1.55	0.3918
Anxiety	1.84 (2.00) ± 1.39	2.90 (3.00) ± 1.23	0.0159*
Stereotypy	1.45 (1.00) ± 1.06	2.07 (2.00) ± 1.03	0.3853
ATEC L	12.16 (12.00) ± 7.49	13.14 (13.00) ± 8.18	0.254
ATEC S	13.64 (15.00) ± 6.31	17.83 (18.00) ± 9.83	0.113
ATEC P	13.68 (13.00) ± 7.77	16.86 (18.00) ± 8.53	0.212
ATEC SC	25.35 (25.00) ± 10.79	27.17 (25.00) ± 11.03	0.119
ATEC T	64.84 (63.00) ± 26.82	75.00 (78.00) ± 32.89	0.098
CARS	33.47 (31.00) ± 8.48	37.83 (39.00) ± 9.02	0.188

CARS = Childhood Autism Rating Scale.

Results are expressed as average (median) ± standard deviation.

All p-values were calculated for the treatment after versus the control after groups using the two-factor mixed analysis of variance (two-way ANOVA) test followed by Tukey and Wilcoxon.

The Autism Treatment Evaluation Checklist (ATEC) subscales are as follows: ATEC L, related to language; ATEC S, related to socialization; ATEC P, related to sensory and cognitive perception; and ATEC SC, related to health, physical aspect, and behavior, while ATEC T, is the total sum of the scale.

* p < 0.01; ^†^ p < 0.05; ^‡^ p < 0.001.

It can be observed from the results of the semi-structured interview that children who received the CBD-rich cannabis extract showed a significant improvement in psychomotor agitation, started to accept more meals a day, had much improved social interaction, and were less anxious, when compared to children in the control group, suggesting improvement in some symptoms associated with the ASD condition. On the other hand, regarding the “concentration” variable, it was observed that only children with mild ASD who received the CBD-rich cannabis extract showed significant improvement in this variable (Table 4). For this reason, it could be suggested that there is a difference between different levels of ASD severity for the “concentration” variable only.

**Table 4:** Mixed analysis of variance for two factors (R software version 4.0.2)

Variable	df	Sum Sq	Mean Sq	F value	Pr(>F)
Social interaction	1	17.63	17.633	14.133	0.000268*
Residuals	116	144.73	1.248		
Psychomotor agitation	1	14.70	14.700	9.225	0.00295†
Residuals	116	184.84	1.593		
Anxiety	1	10.21	10.208	5.989	0.0159^‡^
Residuals	116	197.73	1.705		
Number of meals per day	1	9.63	9.633	4.109	0.045^‡^
Residuals	116	271.99	2.345		
Concentration (mild group)	1	5.56	5.558	6.747	0.0124^‡^
Residuals	48	39.54	0.824		

All p-values were calculated for the treatment after versus the control after groups using the two-factor mixed analysis of variance (Two-way ANOVA) test followed by Tukey and Wilcoxon.

* p < 0.001; ^†^ p < 0.01; ^‡^ p < 0.05.

Four children dropped out of the study during recruitment and laboratory testing prior to the start of the clinical trial. Three of these had been recruited for the control group and one for the treatment group, thus resulting in a final sample of 60 children (31 in the treatment group and 29 in the control group). The reason for dropping out was that they did not live in the city where the clinical trial was being run and had difficulties traveling there. Furthermore, we also analyzed some important hematological parameters, including complete blood test, glycemia, aspartate aminotransferase (ALT), alanine aminotransferase (AST), urea and creatinine, and it was observed that all these parameters were within normal limits in all children.

The Supplementary Material (online-only) presents additional two-way mixed ANOVA statistical analyses.

## Discussion

Even though the CBD-rich extract was used at a low concentration (2.5 mg/mL), using three drops twice a day, and the basic dose was determined by the ABRACE protocol, improvements were observed in social interaction, psychomotor agitation, number of meals, anxiety, and concentration, and the adverse effects experienced by a few of the children were mild and transient. Results showing improved concentration were only observed in the mild group.

The sample comprised 86.7% male children and was not selected by gender, but it is known that ASD is more common in boys than in girls.^5,11^ Females, as a rule, are more rarely affected (four boys to each girl for autism and 10 boys to each girl for Asperger’s Syndrome). This pattern led to the hypothesis of a “female protective effect,” a purported biological aspect by which females require a greater etiological “burden” to manifest autism.^12,13^ Enrollment for possible participation in the clinical trial was open to the general population, but there were significantly fewer female children (296 children enrolled, 45 of whom were female, thus corresponding to only 15.2%, and eight girls [13.3%] and 52 boys [86.7%] were selected for the clinical trial).

Concerning the challenges of the COVID-19 pandemic for people with ASD, given the importance of routine in their lives, families with children with ASD face enormous challenges to mitigate the impact of the condition, as they often fail to carry out preventive measures. Sudden changes, such as social isolation, can cause emotional and behavioral changes, such as anxiety, agitation, and agressiveness.^14^ It is not clear whether COVID-19 had any impact on the study, but differences were observed in medical consultations. For this reason, at the end of the study, parents were asked whether social isolation, which led to temporary discontinuation of multidisciplinary treatments and consequent changes in routine, had interfered with the children’s functioning and 71.7% reported that there was no interference with any significant impairment.

The neuropsychological assessment is crucial to complement a diagnosis of ASD and to monitor the child’s progress while undergoing medication-based interventions. It was also important that neuropsychological tests, such as executive functions, Theory of Mind, empathy, and attention^15^ were performed to evaluate the main psychological functions related to ASD in several areas, such as severity of autism and verbal language.

### Efficacy of CBD-rich cannabis extract

One of the core symptoms of ASD, and one of its DSM-5 diagnostic criteria, is persistent impairment in social interaction. In the present study, the result revealing the most robust improvement (p < 0.001) was social interaction. The reduction in psychomotor agitation is of great relevance. Parents of ASD children often report several food restriction problems and inadequate diet.^16,17^ These issues were observed in our sample, in which the caregivers said that many participants had fewer daily meals than desired and had difficulty eating due to sensory and food selectivity issues. An improvement was observed in eating habits in the treatment group, seen in the parameter “meals.” Anxiety accompanies many children with ASD and can lead to behavioral changes, given that they often cannot express what they feel, and also leads to psychological suffering.^18-20^

Another major complaint of parents of ASD children relates to concentration, so it was very important to analyze this according to the degree of severity of ASD, because mild children possibly have less cognitive impairment, since they require less support, which could suggest that improvement in the ability to concentrate might only occur at this severity level, as was found in this study, but we cannot confirm such a hypothesis on the basis of this clinical trial.

A study with 400 individuals in New Zealand evaluating the prescription of CBD in clinical practice also assessed neurological symptoms, which included Parkinson’s disease, multiple sclerosis, epilepsy, ASD with challenging behavior, amyotrophic lateral sclerosis, multiple system atrophy, chronic pain, various neuropathies, and tremors. Mental health symptoms include anxiety disorders, depressive disorders, post-traumatic stress disorder, stress disorder, and insomnia.^21^ However, to date, there have been no randomized, double-blind, placebo-controlled clinical trials with samples composed only of children with ASD, regardless of stratification by severity.

Although retrospective, one research study with a sample of 60 individuals with an average age of 11 years did show improvements in behavioral outbursts (61%) and anxiety (39%).^22^ A prospective study with cannabis use, which also included adults (53 participants aged 4 to 22 years) and employed biweekly evaluations using structured interviews, resulted in 67.6% improvement in self-injury and bouts of anger, 68.4% improvement in hyperactivity, 71.4% improvement in sleep disturbances, and 47.1% improvement in anxiety, with mild to moderate adverse effects, such as drowsiness and decreased appetite.^23^

In another prospective study, 188 children were observed for 6 months, with all subjects receiving cannabis. From the results of structured questionnaires filled out by their parents, it was found that 30.1% of the subjects presented significant improvement, 53.7% moderate improvement, 6.4% slight improvement, and 8.6% presented no improvement, with agitation (6.6%) and drowsiness (3.2%) as adverse effects.^24^

One observational study looked at efficacy and tolerability over 6 to 9 months, including analysis of comorbidities using monthly structured questionnaires, and found that 93% improved 30% or more in at least one symptom category, 47% improved 30% or more in four or more symptom categories, 13% improved 30% or more in two symptom categories; and 33% improved 30% or more in one symptom category. The symptom categories were as follows: 1) ADHD; 2) behavioral disorders; 3) motor deficits; 4) autonomy deficits; 5) communication and social interaction deficits; 6) cognitive deficits; 7) sleep problems; 8) seizures.^25^

Individuals with ASD who used the CBD-rich cannabis extract showed improvement in the following symptoms: self-injury and bouts of anger, hyperactivity, sleep problems, anxiety, restlessness, psychomotor agitation, irritability, aggressiveness, sensory sensitivity, cognition, attention, social interaction, language, perseveration, and depression. Regarding the benefits of the intervention with cannabis, the restlessness symptom showed the greatest improvement (91%) in relation to the other symptoms studied.^26^

Therefore, our results show what the scientific literature demonstrates about its efficacy in hyperactivity, restlessness, and psychomotor agitation; in anxiety; in cognition, attention and concentration; in facilitating learning; in nutrition; and in social interaction in children on the autism spectrum.

### Safety of CBD-rich cannabis extract

Currently, CBD is approved by the Food and Drug Administration (FDA) for treatment of Dravet and Lennox-Gastaut syndromes, which are related to seizures.^27^ Randomized clinical trials have shown that when CBD is added to an anticonvulsant, the frequency of seizures decreases.^28,29^ As the scientific literature shows good response to epilepsy, we included children who presented epilepsy as a comorbidity as an exclusion criterion, in order to specifically analyze the characteristics of cannabis for ASD.^30,31^

Numerous pre-clinical studies^32,33^ and neuroimaging studies^34^ have demonstrated the anxiolytic effects of CBD. A published case series in psychiatric patients found CBD was beneficial for anxiety and sleep.^35^ According to the results described, anxiety is identified as a relevant characteristic associated with ASD, corroborating the scientific literature that presents overall improvement in anxiety in some studies.^32-35^

Researchers advise medical professionals, who encounter young patients using CBD, to discuss its quality and possible adverse effects and drug interactions, which were carefully analyzed in this study. If any subject had exhibited poorly understood symptoms such as fever, diarrhea, vomiting, or drowsiness, the adverse effects of CBD oil would not have gone unnoticed.

In this trial, it was found that only three children in the treatment group (9.7%) had adverse effects, which were dizziness and insomnia in one child, colic in one, and weight gain in another. In some studies, the following symptoms of adverse effects were observed: sleep disorders, restlessness, and nervousness, as well as moderate irritability, diarrhea, increased appetite, conjunctival hyperemia, behavioral problems, decreased cognition, fatigue, and aggression/agitation.^24,25^

These agents need to be evaluated over time for the long-term effects of these drugs on development, which remains an open question.

One of the limitations of the study was the coronavirus pandemic, which started during the clinical trial. Since routine is crucial in the lives of children with ASD, their families were faced with enormous challenges to mitigate the impact of the condition. As the children were divided into six groups of 10 for consultation and product initiation, there was a difference regarding the start of use: those who started before the pandemic (COVID-19) and those who started during the pandemic, since the clinical trial started in January 2020 and a countrywide quarantine was implemented in March of the same year in Brazil. For the same reason, laboratory tests could not be performed after the end of the clinical trial.

New randomized, double-blind, placebo-controlled clinical trials using CBD-rich cannabis extract at higher concentrations and even using isolated CBD (phytochemical) for similar analyses would be an important contribution.

## Conclusion

The CBD-rich cannabis extract was found to be safe at the doses used in this study (ranging from six to 70 drops/day), given that only three of the 31 children who received the extract reported very mild side effects, such as dizziness, insomnia, colic, and weight gain. Titration could reach a maximum of 100 drops per day, as directed by the product supplier.

Based on the results obtained, it can be concluded that CBD-rich cannabis extract showed significant improvement in social interaction, anxiety, and psychomotor agitation when compared to children who received the placebo and that CBD-rich cannabis extract did not interfere with the children’s sleep quality.

Another important result in this study was an increased number of meals per day among children who received the CBD-rich cannabis extract when compared to the children who received the placebo. This result may be related to the decreased anxiety levels of these children observed after administration of the extract.

Therefore, it is observed that CBD-rich cannabis extract is effective and can be used safely, at least in the short term, to relieve some important symptoms related to ASD in children, such as social interaction, psychomotor agitation, and anxiety.
