# Supplementary material for: Evaluation of the efficacy and safety of cannabidiol-rich cannabis extract in children with autism spectrum disorder: randomized, double-blind, and placebo-controlled clinical trial
Source: Trends Psychiatry Psychother. 2024 Feb 28;46:e20210396. doi: 10.47626/2237-6089-2021-0396 (PMC11332686; doi:10.47626/2237-6089-2021-0396)
Supplement: Supplementary file 1 [file 2238-0019-trends-46-e20210396-suppl.pdf]

http://doi.org/10.47626/2237-6089-2021-0396

Two-Way Mixed ANOVA (Psychomotor Agitation)

	Df	Sum Sq	Mean Sq	F value	Pr(>F)
Time	1	14.70	14.700	9.225	0.00295 **
Group	1	2.99	2.991	1.877	0.17334
time:group	1	0.94	0.937	0.588	0.44465
Residuals	116	184.84	1.593		

---Signif. codes: 0 ‘***’ 0.001 ‘**’ 0.01 ‘*’ 0.05 ‘.’ 0.1 ‘ ’ 1

Two-Way Mixed ANOVA (Number of Meals)

	Df	Sum Sq	Mean Sq	F value	Pr (>F)
Time	1	9.63	9.633	4.109	0.045 *
Group	1	4.24	4.236	1.807	0.182
time:Group	1	0.01	0.011	0.005	0.946
Residuals	116	271.99	2.345		

---Signif. codes: 0 ‘***’ 0.001 ‘**’ 0.01 ‘*’ 0.05 ‘.’ 0.1 ‘ ’ 1

Two-Way Mixed ANOVA (Social Interaction)

	Df	Sum Sq	Mean Sq	F value	Pr (>F)
Time	1	17.63	17.633	14.133	0.000268***
Group	1	4.49	4.490	3.599	0.60300
time:group	1	0.35	0.349	0.280	0.597960
Residuals	116	144.73	1.248		

---Signif. codes: 0 ‘***’ 0.001 ‘**’ 0.01 ‘*’ 0.05 ‘.’ 0.1 ‘ ’ 1

Two-Way Mixed ANOVA (Anxiety)

	Df	Sum Sq	Mean Sq	F value	Pr(>F)
Time	1	10.21	10.208	5.1989	0.0159*
Group	1	6.87	6.872	4.031	0.0470*
time:group	1	0.51	0.512	0.300	0.5847
Residuals	116	197.73	1.705		

---Signif. codes: 0 ‘***’ 0.001 ‘**’ 0.01 ‘*’ 0.05 ‘.’ 0.1 ‘ ’ 1. **(Time: Before or after / Group: treatment or control)**

Two-Way Mixed ANOVA (CONCENTRATION – MILD SEVERITY)

	Df	Sum Sq	Mean Sq	F value	Pr(>F)
Time	1	5.76	5.558	6.747	0.0124 *
Group	1	2.33	2.327	2.825	0.0993
time:group	1	1.56	1.558	1.891	0.1755
Residuals	48	39.54	0.824		

--- Signif. codes: 0 ‘***’ 0.001 ‘**’ 0.01 ‘*’ 0.05 ‘.’ 0.1 ‘ ’ 1. **(Time: before or after / Group: treatment or control).**
